# What About Us? Experiences of Relatives Regarding Physician-Assisted Death for Patients Suffering from Mental Illness: A Qualitative Study

**DOI:** 10.1007/s11013-021-09762-1

**Published:** 2021-12-16

**Authors:** Rosalie Pronk, D. L. Willems, S. van de Vathorst

**Affiliations:** 1 Department of Ethics, Law and Humanities, Amsterdam UMC, Academic Medical Centre, Room J2-126, PO Box 22660, 1100 DD Amsterdam, The Netherlands; 2 Department of Medical Ethics and Philosophy, Erasmus Medical Centre, Rotterdam, The Netherlands

**Keywords:** Physician-assisted death, Mental illness, Relatives, Family members, Netherlands

## Abstract

Physician-assisted death (PAD) for patients suffering from mental illness is legally permitted in the Netherlands. Although patients’ relatives are not entrusted with a legal role, former research revealed that physicians take into account the patient’s social context and their well-being, in deciding whether or not to grant the request. However, these studies focussed on relatives’ experiences in the context of PAD concerning patients with somatic illness. To date, nothing is known on their experiences in the context of PAD concerning the mentally ill. We studied the experiences of relatives with regard to a PAD request by patients suffering from mental illness. The data for this study were collected through 12 interviews with relatives of patients who have or had a PAD request because of a mental illness. We show that relatives are ambivalent regarding the patient’s request for PAD and the following trajectory. Their ambivalence is characterised by their understanding of the wish to die and at the same time hoping that the patient would make another choice. Respondents’ experiences regarding the process of the PAD request varied, from positive (‘intimate’) to negative (‘extremely hard’). Some indicated that they wished to be more involved as they believe the road towards PAD should be a joint trajectory. To leave them out during such an important event is not only painful, but also harmful to the relative as it could potentially complicate their grieving process. Professional support during or after the PAD process was wanted by some, but not by all.

## Introduction

Dutch law has regulated the practice of physician-assisted dying since 2002. Physicians may be exempted from prosecution if, and only if, they meet the legal criteria of due care. Relatives of patients who request PAD have no legal role before, during or after the process. We do, however, know that family members can be active participants in the decision-making process of PAD in case of somatic diseases. In general, the family of the patient plays an important role for physicians when deciding whether to grant a PAD request (Roest, Trappenburg and Leget 2019, Snijdewind et al. 2014). The well-being and future bereavement of the family members are often taken into account when physicians decide whether to grant the PAD request or not (ten Cate et al. 2017). Whether relatives should be involved during a PAD trajectory is debated. Various authors claim that the practice of PAD needs to be understood in terms of a patient-physician-family triad, instead of just the patient and the physician, because physicians often already engage relatives anyway. (Roest et al. 2019; Snijdewind et al. 2014) On the other hand, physicians need to make sure that the request is not made under the undue influence of relatives, so that the request can be considered voluntary (EuthanasieCode 2018). Research shows that more than one-third of Dutch physicians feels pressured by family members of the patient to perform PAD (in case of somatic illness) (Onwuteaka-Philipsen et al. 2017). This does not necessarily imply that the patient is also pressured into PAD, but it does show that family members can influence the evaluation of a PAD request in general.

The majority (90.6%) of PAD requests are made by patients whose suffering is caused by a somatic illness, such as cancer or cardiovascular disease (Jaarverslag 2020). The Dutch euthanasia law, however, does not exclude patients who suffer from mental illness. In the year 2020, out of all 6938 reported PAD cases in total, 88 were cases that involved patients suffering from mental illness (Jaarverslag 2020). PAD requests on the grounds of suffering from a mental illness are mostly performed by Expertisecentrum Euthanasie (EE) (formerly known as the ‘End-of-life Clinic’) (Kammeraat and Kolling 2020; Jaarverslag 2019) They often receive the more complex requests, and patients for whom a mental illness is the underlying reason for requesting PAD are considered complex by physicians (Bolt et al. 2015; Evenblij et al. 2019a, 2019b).

Empirical research in which the relatives participate as a research subject are relatively scarce (Roest et al. 2019) and former studies on relatives and PAD mainly focussed on relatives of patients who suffer from somatic illnesses, revealing that these relatives are often involved during PAD trajectories. It is likely that also in the case of PAD for mental illness, relatives are involved. As the number of PAD cases involving patients with mental illness is rising (Onwuteaka-Philipsen et al. 2017), and the Dutch guideline on how to adequately assess PAD requests from the mentally ill states that relatives should be involved as much as possible in all phases of the trajectory (https://richtlijnendatabase.nl/richtlijn/levensbeeindiging_op_verzoek_psychiatrie/startpagina_-_levensbe_indiging_op_verzoek.html), it is important to understand what the experiences are of relatives of patients suffering from mental illness who have a PAD request. Therefore, we set up this qualitative study to examine the experiences of the relatives of patients that requested PAD because they suffer from a mental illness. In this study, the term ‘relatives’ refers both to family members, and also to close friends of the patient.

## Methods

We chose to address the questions by using qualitative methods, as this would provide the best opportunity for respondents to elaborate on their experiences and would lead to rich data.

We held 12 interviews with relatives from patients who had requested PAD because of their suffering from a mental illness. These relatives related to 12 unique PAD cases, which means we did not interview multiple persons related to one PAD case. These interviews were held between June 2019 and June 2020. The interviews had an open character but were structured by the use of a topic list, with topics such as: how do they experience the death wish of the patient, how does the PAD request impact them, do they support the PAD request and why, how do they experience the procedure of the PAD request, are they involved during the procedure and how do they experience this (lack of) involvement, what do they think about how the physician handled the PAD request, how do they feel about the decision of the physician, are they in need of support during or after the trajectory, where would they like to find this support?

One researcher (RP) conducted all the interviews. Because of the situation around Covid-19, 3 interviews were held by video call, but the remaining 9 could be held face-to-face.

The interviews were held at the location of the respondent’s choice and lasted between 1 and 3 h. Respondents signed an informed-consent form, that included statements on confidentiality and the voluntary character of participation. We also promised confidentiality on information given about the patient and his situation. The respondents who were interviewed via video call were read the informed-consent form and agreed verbally.

This study did not need ethical approval of an ethics committee under Dutch law (WMO).

### Respondents

We recruited the respondents through the Dutch Dying with Dignity foundation (NVVE). We wrote a call, that was published in the newsletter of the NVVE, to which potential respondents could respond. These relatives had actual experience with their relative requesting PAD, so for them it was not just a hypothetical situation. Unfortunately, we could not find more than 12 respondents that wanted to give an interview. Characteristics of the respondents can be found in Box 1. One person was not interviewed, but responded through email about the PAD of her son. Passages of this email were also used in this paper.

**Box 1:** Respondent characteristics

Respondent number	Sex	Relation to the patient	Status at the time of the interview
R1	Male	Friend	Patient received PAD
R2	Male	Partner	Patient still in process of PAD
R3	Female	Mother	Patient received PAD
R4	Male	Brother	Patient still in process of PAD
R5	Male	Brother	Patient received PAD
R6	Female	Partner	Self-inflicted death of patient
R7	Female	Friend	Patient received PAD
R8	Male	Partner	Patient received PAD
R9	Male	Partner	Self-inflicted death of patient
R10	Female	Mother	Patient still in process of PAD
R11	Female	Mother	Self-inflicted death of patient
R12	Female	Friend	Patient still in process of PAD

### Data Analysis

All interviews were transcribed verbatim by a third party, who had signed a confidentiality statement. Analysis of all interviews was done by RP, and methods and results were discussed with the supervisors of the study (SV and DW). We used open coding and inductive analysis to identify overarching themes. The codes were based on the content of the interviews. Analysis was supported by the use of a qualitative coding programme: MAXQDA2020.

## Results

In the results, we will discuss the following themes: when a relative wants PAD, the process of requesting PAD, and support for the relatives.

### If a Relative Wants PAD

Although the interviewed relatives eventually supported the decision of the patient requesting PAD, they often did so with ambivalent feelings and their support was often the result of a process. Respondents mentioned various aspects of their process; they understand the wish of the patient, but also hoped for another solution. Understanding the suffering of the patient, or ‘compassion’, was an aspect that the respondents often mentioned. They saw how hard life was for the patient and how deep their suffering was. They understood that this was just not bearable anymore for the patient: they wanted the patient to have peace. This respondent describes the ambivalence of understanding the wish to die on the one hand, and wanting to grow old with the patient on the other:

‘And I understood her suffering, I understood her burden and wish, but I wanted to grow old with her. We fought some fights about that during the summer. (…) After that, we sort of went on a two-track approach, she went on the track of the Expertisecentrum Euthanasie and I went on the track of wanting to keep her. So, ‘I will follow, but I won’t go along’…and that led to more friction and clashes. So eventually I had to make the choice, either I keep doing this and I lose her, or I get beside her and walk with her, still hoping I could turn her. I did the last thing. But that got me in an exceptional position. (…) During that time she informed a couple of people, some friends, who had all kinds of questions about it and came to me with those questions. And I had to defend her, or explain her, while I did not want this to happen at all.’ R6-partner (self-inflicted death of patient).

In some cases, the respondents understood the wish to die from their own experience, as they also suffered from a mental illness and had had periods in which they wished to take their own life. They explained that their own experience with wanting to end their life as a result of a mental illness contributed to a deeper understanding of why the patient wished to end their suffering, but also led to their hope that the patient would also be able to recover. Respondents on the one hand understood the patient’s wish to end their life, but they feared that they would do so by means of a gruesome suicide. Respondents wished that the patient did not have to die because of suicide, because they wanted them to have a humane and peaceful death, also without possible legal consequences for the relatives.

Finally, some respondents spoke about their belief that the patient should be able to make decisions about their own life, and that the respondent thought that the patient was capable of doing so. Some respondents believed the patient still had potential to recover from their mental illness. They believed that more or a different treatment for the patient’s mental illness would result in the patient wanting to continue his life. Another aspect of the struggle with the wish to die of the patient was related to religious beliefs of the respondent. This respondent for example speaks about how his Christian beliefs do not allow for a self-chosen end-of-life:

‘And I come from a Christian background. The idea to end her life, actively end her life, was not done in our family culture. From the perspective of the Bible…well, you get a death sentence when you try to end your life. And people who end their own life, they cannot be buried in the cemetery, they used to be buried next to the cemetery. (…) It is sort of a desecration of the ground if you end your own life. So that cultural, or religious determination, or the little space that you have as a human being…whether that is because you are gay, or mentally ill, if you could not bear life anymore and would take your own life, you were condemned to an eternal burning in hell, so to speak.’ R5—brother (patient received PAD).

### The Process of Requesting PAD

All respondents were involved with the patient’s request for PAD in one way or another. Some respondents only discussed the PAD wish and request with the patient. Others were also present during conversations with the physician. Some were even present at the moment that the patient died. Not all respondents were satisfied with the extent to which they were involved in the PAD process; some mentioned how they were not present during conversations with physicians even though they had wanted to be, because they wanted to understand how the patient and physician viewed the situation. Respondents who were friends with the patient understood why they were not involved in a way that for example a partner or mother was. Not all relatives were asked to be part of the trajectory by the patient, e.g. because the patient and relative had a disturbed relationship (e.g. very little contact) Not being a part of the conversations between patient and physician was problematic for some respondents, because they felt they could offer valuable information about the situation and wanted to express their view on the matter. Also, being part of the process was considered by the respondents to be better for the relative, because they believe that it should be a joint trajectory.

Some respondents felt left out by the physicians of Expertisecentrum Euthanasie, even though they were present at the conversations the physician had with the patient:

‘I: And do you have the feeling that you were involved in the process?

R: By her: yes. Not by Expertisecentrum Euthanasie. I was there during all conversations, except that one conversation with her own psychiatrist, all other conversations were here at home, so I was present at all of them.

I: okay.

R: But that is also a thing of the team [i.e. of Expertisecentrum Euthanasie], I understand it at the one hand, but not on the other. They are here for the patient, and not for the relatives that go with that. That is the idea that they gave me.

I: And how did you feel about that?

R: I cannot judge whether this is how it normally goes, or just with this team. (…) Let’s say that I can imagine that for some survivors it would be nice. Yes. (…) would I have wanted that? Yes, I actually do. It is still something that you do together, this trajectory.’ R8 – partner (patient received PAD).

‘I: Did you feel involved by Expertisecentrum Euthanasie?

R: No, No. I goes past you. They never asked us anything. Partly, I understand it, because it has to revolve around the one making the request, but I do think ‘well, it is a road that you walk together’, in the best case of course. (…) If you ask me, I understand that it goes like this, but I think that for the survivors or relatives it would be better if it is a more joint trajectory, if that is possible within the family ties. R3 – mother (patient received PAD).

Some patients made the choice to keep the PAD request secret from relatives, or asked one relative to keep the PAD request a secret from another relative. Respondents mentioned how keeping the PAD request secret from other relatives can provoke feelings of unsafety amongst families:

‘Eventually after two years, I was able to accept it, and my brother was also able to accept it, but my sister was very troubled by it. And my other brother, who was very much against it, believed it [i.e. PAD] just should not have happened. So everyone had their own position. But fact was that it was a huge blow for everyone, that was unbalancing for all of them. And I mean, people who consider this [i.e. PAD] should not keep it a secret for so long, because it is horrible for the relatives, it undermines your feeling of safety. You can just hear out of the blue that your brother or sister is going to die a month later…and is getting help doing so, the Dutch government, mental healthcare facilities are helping with that. ‘it should not get any crazier’ those thoughts are present. If you are not part of that process (…) if you do not do that, it is a blow for the relatives that leaves them insecure for a very long time. R5—brother (patient received PAD).

A lack of involvement was not always perceived as problematic, as some respondents indicated that it is the patient who can make the decision about whether and how their relative should be involved, and not them. It depended on the relationship with the patient whether the respondent found this problematic or not. For example, friends did leave more room for the personal decision of the patient. Family or partners were more inclined to be troubled by a lack of involvement.

Some respondents experienced the process of requesting PAD and the trajectory with the physician as hard, lonely or bizarre. One mother wrote to us in an email (she was not interviewed) that it took a lot from her and her child to stay alive until the PAD was performed:

‘Everything around the self-chosen end-of-life of R. [i.e. name patient] was as he wanted it, but the road towards it was extremely long and hard. There were moments in which R. almost could not hang on any longer and we had to do everything with help, care and support with medication to make sure he would not hurt himself. It was literally keeping someone alive so that he could die with dignity.’ *–* mother (patient received PAD).

And a partner said:

‘And the whole trajectory, well, I think it is bizarre. It is bizarre to stand next to someone who is on a road to death, and longs for death. Who during the process (…) gets a very narrow view, while I did not want to lose her.’ R6 – partner (self-inflicted death of patient).

Other respondents described the PAD trajectory in positive terms, such as this mother, who experienced the process as ‘intimate’:

‘R: And you know, it is the worst thing that can happen to you as a parent, and yet I consider myself lucky that it happened like this. I went through worse grieving processes. I mean, I am not cheering, and it hurts on a daily basis, but I do not want to miss that pain anymore, it is part of it [i.e. her life] now, and it is bearable out of love. It makes you a different person, I hope a better person, I do not know.

I: So it was also a positive experience, even though it was really hard?

R: Yes, absolutely, yes. Very intimate.’ R3—mother (patient received PAD).

### Support for the relatives

Some relatives experienced a sense of burden in having to care for their loved one. Although they loved the patient a lot, and did not wish for him to die, they considered the care to be burdensome. This respondent explains how he experienced less stress and more freedom now that the patient has passed away:

‘And I was in peace with that. I thought, if she dies, that will also be some sort of relief, because I also lose a lot of stress and care. I have more freedom to do things again, until Corona happened, because now I cannot go anywhere.’ R9 – partner (self-inflicted death of patient).

During the process of PAD, they received support mostly from people in their surroundings. This respondent mentioned how he finds support with friends and neighbours:

‘I have good friends in W. [i.e. town] and we have very nice neighbours, they listen to us, offer us things. But I would not have the slightest idea what people could have done. No one can fix the head of M. [i.e. name patient]. So, yes, support, like last Sunday, we went to a good friend of M., we had a reasonable day. Just some distraction, that is the support I need, yes.’

#### R2—Partner (Patient Still in Process of PAD)

Some found professional help, from for example their general practitioner or a mental healthcare provider. In some cases, the mental healthcare provider of the patient also was a source of support for the relative. They appreciated this a lot, also because the care provider knew the patient.

We asked whether the relatives were in need of more support during the process of requesting PAD, perhaps also organised in a more structured way; for example by means of peer-support. The respondents reacted differently to this idea: some would have liked this; others did not want this at all. Reasons for wanting an organised structure were that they could find recognition and information from others. On the other hand, respondents indicated that peer-support would be too much, as they were still taking care of the patient. Also, as the patient was so well-informed about the trajectory, they could share the information with their relative, hence there was no need for more information.

We asked the respondents whether they received some type of professional support after the patient died, whether they would have liked to receive support, and in what form. Most respondents mentioned that they sought support with friends and family. Some respondents indicated that they sought psychological treatment themselves, or participated in peer-support groups with a focus on grief (not necessarily related to PAD).

For some respondents, finding support with friends and family was enough, but some were in need of more support. This respondent for example talks about how his brother-in-law (the partner of the patient) would have liked to hear something from the treating physician of the patient:

‘R: There is one thing I have noticed, not during the process, but after it ended. The relatives, my brother-in-law never heard anything, never, not a phone call, no visit, nothing.

I: From the physicians, you mean?

R: From the treating physician, who treated his wife for 15 years.

I: Okay, yes.

R: While he walked on eggshells for years, always with a fear of ‘is she still there?’ and ‘how is she?’. So it was horrible for him, his wife that he loved, that he was married to for 30 years, to lose her like this. (…) And a sign of sympathy from the mental healthcare system, yes, he needed that, and he missed that a lot.

I: Yes.

R: Why did they not reach out to him? That is the only thing that I can think of: ‘professionals should pay more attention to that, the aftercare’’ R5 – brother (patient received PAD).

We also asked whether a peer-support group for the moment after the PAD was performed would be an option for the respondents. For some, a peer-support group would not be an option, as they indicated that they do not want to listen to other people’s stories. So, although peer-support groups are not for everyone, some liked the idea of help with overcoming or coping with the grief and loss of the patient. This respondent spoke about how she would like to have support to fill the void and create some order in the chaos that the death of the patient leaves:

‘I: And what would aftercare mean to you, what are you in need of?

R: Structuring, structuring within the context of…well, structuring the chaos. And how it is best arranged, I have to think about that. But this is something really intense, whether it [i.e. PAD) happens or not. It is a measuring moment, that is how I see it, a milestone that absorbs everything. And after that, there is nothing, nothing, just chaos. And I really look up to that. (…) it concerns me. I think ‘yes’, I would like that [i.e. aftercare). I think that is a good thing. But I cannot think of something other than a black hole, and the chaos. That it [i.e. aftercare] is important in some form or another.’ R10 – mother (patient still in process of PAD).

## Discussion

We performed this study to investigate the experiences of relatives of patients with a PAD request as a result of suffering from a mental illness. We show that the process of requesting PAD and the following trajectory comes with ambivalence. It involves understanding the wish to die, but at the same time hoping that the patient would make another choice. Aspects of understanding the wish to die were compassion, respect for autonomy, having experience with the same mental illness as the patient, and the fear that the patient will commit a gruesome suicide. On the other hand, respondents had personal beliefs (such as religious beliefs) or beliefs about the situation of the patient (perhaps they can get better with more or different treatment?) that conflicted with understanding the PAD request. Respondents’ experiences regarding the process of the PAD request varied, from positive (‘intimate’) to negative (‘extremely hard’), or a combination of positive and negative feelings. These results may be relevant for other areas in the world where either PAD for patients suffering from mental illness is already permitted (such as Belgium), or heavily debated (Canada).

One of the results that stood out was the ambivalence all respondents experienced with regard to the PAD request; not one simply just agreed with the wish of the patient. They sometimes experienced conflicting emotions or values, such as understanding the wish on the one hand versus having religious beliefs that do not comply with this feeling on the other. This ambivalence is a known phenomenon, that is also present in the context of PAD for patients suffering from somatic diseases.(Roest et al. 2019) We found that the quality of the ambivalence experienced by our respondents is similar to the quality of the ambivalence experienced by relatives of patients who request PAD as a result from suffering from a somatic illness, as Roest states: ‘while they wish for the patient’s suffering to end, and regardless of personal views on EAS, they often considered EAS to be too early, or too definitive’.(Roest et al. 2019) However, although respondents indicated that they felt ambivalent, they did not mention that they suffered from this ambivalence. What they did suffer from was that the physician and patient did not take their views into account when evaluating the request. Some respondents did not understand why the physician would help the patient to die, and were very much against it; they were not part of the trajectory. Keeping the PAD a secret from other relatives meant a huge emotional blow for all involved. It seems important to some relatives that the PAD is a joint trajectory.

This relates to a second important result of our study: although most respondents were involved in the process in one way or another, some of them were not satisfied with how much or the way they were involved. Multiple respondents mentioned that they did not feel engaged by EE during the process. Although they understood why not, they did wish for more involvement, as they preferred that the process towards PAD should be a joint trajectory. We asked EE what their policy is on involving relatives when evaluating PAD requests. Their statement can be found in Box 2.

**Box 2:** Statement from Expertisecentrum Euthanasie (EE)

EE aims to work carefully and with due diligence for the patient and their family members. EE also preferably works with the patient’s general practitioner and (in case there is one) with the patient’s treating psychiatrist. Although EE preferably involves the relatives, the patient’s wish is leading. This means that if the patient decides that he/she does not want to involve relatives, this wish is respected. In the case relatives are involved, EE asks them for their opinion and solicits their views on the subject, but relatives have no say in the decision to grant the request or not. If relatives were involved, EE calls them about 6 weeks after the PAD is performed as a form of follow up care	

So, although EE explicitly has the intention of involving the relatives of the patient during the PAD process, and all relatives were involved in one way or another, our results show that relatives sometimes do not *feel* involved (enough). Engaging relatives in the PAD process seems to have a positive effect on the grieving process of the relatives (Dees et al. 2013; Norwood 2009) General practitioners and nursing home physicians often do so, and physicians in general describe a positive experience with involving the family members during a PAD process in context of somatic illness (Muller et al. 1996; Kimsma and van Leeuwen 2007). On the basis of our study, we could say that more awareness should be raised on how to make relatives feel included. We need to gain more insight in what exactly makes relatives feel left out, what they would need to feel included and how to facilitate that inclusion. This study alone does not provide enough answers to that question. Some of these answers can be found in the publication of a Dutch study that was conducted in which they held focus groups meetings with family members of patients who had a PAD request due to suffering from a mental illness (Ettema et al. 2019). Results show that family members want physicians to involve them during the process and show that they understand their situation, that they discuss expectations and wishes of relatives, use the knowledge and expertise of relatives, and support and attend to after care and evaluate the process together with the relatives. These findings resulted in a checklist for physicians on how to evaluate a PAD request from a mentally ill patient. The results from these focus group meetings in combination with our results, show the importance of including relatives in the process.

Expertisecentrum Euthanasie (EE) – that handles most requests from patients suffering from mental illness – assumes no task in taking care of the relatives for a longer period of time.These physicians are not the treating physician of the patient and this may complicate caring for the relatives. We expect similar patterns in other countries: even if PAD for persons suffering from mental illness is allowed by law, practice will show that most physicians are reluctant to evaluate and perform such requests (Onwuteaka-Philipsen et al. 2017). If a country does not have an institution like EE to handle requests, it is possible that it comes down to a few physicians who are willing to grant those requests. Although EE calls the relative 6 weeks after the PAD is performed, they do not support the relative during or after the trajectory. General practitioners, however, often also care for relatives of patients, as they are also a patient in their practice. This could be an argument for PAD trajectories by the general practitioner; relatives could perhaps be better cared for by their physician. It would then be the treating physician’s responsibility to organise care or support for the relatives. Another option is to look into a Belgian initiative where peer-support groups, also for relatives, have been established (see www.reakiro.be). To our knowledge, there are no studies available yet on what the experiences of Reakiro or the participating relatives are with regard to these peer-support groups. However, the potential of having a facility where patients, relatives and physicians can come for support, information or recovery-oriented care is probably very important. In the Netherlands, there is no such facility available yet, but we are currently exploring the option of setting up a similar initiative.

Our study shows that relatives want to be taken into account when the patient’s wish is evaluated by a physician. It is not so much that they want to impose their views on patient and/or physician; they want to be acknowledged and to be a part of a trajectory. This is an understandable wish, since relatives often have been part of the patient’s life for a long time (sometimes even from birth) and care for the patient a lot. To leave them out during such an important event is not only painful, but also harmful to the relative as it could potentially complicate their grieving process. In end-of-life care generally, bereavement care of the family at the end-of-life is an important aspect (Mistiaen et al. 2014; Lynn and Adamson 2003). It is conceivable that a stronger involvement of relatives exacerbates a possible conflict between respect for autonomy and beneficence/non-maleficence, because it may be seen to add weight to the autonomy of the patient. So, a physician may feel more forced to follow the request. Related to justice, patients who do not have (active) relatives, may be disadvantaged because they lack the possibility to be fully (i.e. relationally) autonomous. Thus, the main question that arises is whether one should have a relational or non-relational view on autonomy in matters of medical decision-making such as PAD. A non-relational way of understanding autonomy focuses much on independence and control, whereas a relational way includes an understanding of a patient’s personal views and relationships. (Donchin 2000; Ho 2008). In light of this last view, respect for the wishes of the patient could also implicate respecting the wishes of the relatives, as they are often connected. In that light, one could argue that physicians also need to take the relatives into account when confronted with a PAD request: it is part of taking care of the patient, and also in line with the principle of ‘beneficence’ (doing good) for the relative. However, involvement of relatives can also have adverse consequences; what if the patient and his relatives have a conflict-filled relationship and they want to exclude them from the trajectory, of what if the wishes of the patient contradict the views of the relatives? Then, a non-relational view on autonomy seems to be the more appropriate choice. EE seems to have found a more pragmatic approach, in which they see the need of the relatives and try to include them in the trajectory (in line with beneficence and a relational view on autonomy), but also respects the patient’s wish in case they do not want the involvement of others (a non-relational view on autonomy). In line with EE’s view and the Dutch guideline on PAD request from the mentally ill, we would argue that, in case of overlapping views and if the patient wishes so, relatives should be included in the trajectory. If the patient wishes others *not* to be a part of the trajectory, then that wish should be respected. Persons may make autonomous decisions about their end-of-life and about who they wish to be a part of it. Although unfortunate and undesirable from the perspective of the relatives, in the end it is and should be a patient-physician affair.

Another matter related to care for relatives after the PAD trajectory. Taking care of relatives after the PAD trajectory could be a task for the general practitioner, or another professional care giver. However, most respondents relied upon friends and family for support. Also, follow up care is not necessarily medical care. Perhaps it would therefore be better suited to not rely upon general practitioners or other care professionals for this care, but to organize support groups for relatives; as some mentioned that they could benefit from this. It would be greatly appreciated by some of our respondents if there would be a peer-support group especially for relatives of patients that have a PAD request because they suffer from mental illness.

### Strengths and limitations

A strength of the study is that this is the first time relatives of patients with mental illness and a PAD request have been investigated. Other studies mainly focussed on relatives of patients with somatic illness. We succeeded in finding these relatives and getting them to speak openly about this emotionally charged issue. Another strength is that we conducted a qualitative study, with in-depth interviews, which provided respondents to elaborate on their views and experiences. A final strength is that all the respondents had actual experience with a patient requesting PAD, so they did not discuss hypothetical situations.

A limitation of the study is that we only found relatives through the Dutch Dying with Dignity Foundation (NVVE). This may have led to selection bias, as members of the NVVE often have a positive attitude towards PAD in general. However, we managed to also find relatives opposed to PAD for mental illness, for example, because of religious beliefs. We, therefore, believe that we were able to also show results of respondents who have a wider range of attitudes.

Another limitation is the relatively low number of respondents (https://richtlijnendatabase.nl/richtlijn/levensbeeindiging_op_verzoek_psychiatrie/startpagina_-_levensbe_indiging_op_verzoek.html). Although this is inherent to qualitative study designs and it was also expected due to the sensitivity of the topic, it potentially could have led to a lack of data saturation. We acknowledge this risk, but would like to add that we do believe that this study is valuable because it is quite a difficult group to identify and recruit for study purposes due to the relatively low number of patients requesting PAD for suffering from mental illness. As mentioned, it is also understandable that people are hesitant to participate in such a study, because it is a sensitive and emotional topic.
